# Supplementary material for: Substitute for polyethylene (PE) films: A novel cow dung-based liquid mulch on silage cornfields
Source: PLoS One. 2022 Jul 13;17(7):e0271273. doi: 10.1371/journal.pone.0271273 (PMC9278788; doi:10.1371/journal.pone.0271273)
Supplement: Attachment — Submitted filename: Manuscript file.docx [file pone.0271273.s008.docx]

**Substitute for polyethylene (PE) films: a novel cow dung-based** **liquid mulch on silage cornfields**

Xiangjun Yang^a,b┼^, Lu Li^a┼^, Wuyun Zhao^a,*^, Xuan Li^c^, Yongsong Mu^d^, Maohan Chen^b^, Xiaoqiang Wu^b^

^a^ *Faculty of Mechanical and Electrical Engineering, Gansu Agricultural University, Lanzhou 730070, China*

^b^ *Faculty of Mechanical Engineering, Chengdu University, Chengdu 610106, China*

^c^ *Faculty of Water Conservancy and Hydropower Engineering, Gansu Agricultural University, Lanzhou 730070, China*

^d^ *Huarui agricultural company, Zhangye 734500, China*

^┼^: These authors are co-first authors of this work.

**Correspondence**

Wuyun Zhao, Faculty of Mechanical and Electrical Engineering, Gansu Agricultural University,

Lanzhou 730070, China.

E-mail: [zhaowy@gsau.edu.cn](mailto:zhaowy@gsau.edu.cn)

Phone/Fax: +86 0931 7632472

**ABSTRACT**: To lessen the soil pollution caused by non-degradable PE films and rationally dispose animal husbandry waste, the cow dung-based liquid mulch (CDLM), as an alternative to PE films, has been prepared and applied in the central region of the silage corn plots under mulched drip irrigation in Gansu, China. The effects of CDLM were compared with the plots covered by PE films, and non-mulching films as the control (CK) in successive years (2018-2020). The results showed that the CDLM exhibited comparable performance to that of PE films for large-scale planting on keeping soil temperature, silage corn yield, and water use efficiency (WUE). The CDLM presented a complete degradation within 60~100 days, covering the whole vegetative stage of the silage corn growth. Due to the degradation, the CDLM exhibited a super advantage for crop growth and improving soil fertility. Therefore, the CDLM as a potential alternative to PE films was suggested.

***Keywords:*** cow dung-based liquid mulch; polyethylene (PE) films; silage corn; soil organic matters; degradation;

1. **Introduction**

Film mulching techniques are widely used in agriculture to ensure and increase the crop yield, particularly for the areas which are dry, lack rainfall, and with significant temperature differences. PE films, as a kind of typical [mulching](javascript:;) [film](javascript:;)s, have been proved effective in the conservation of soil water, regulation of soil temperature, control of the weed, and improvements in WUE and the yield [1-3]. However, despite the positive effects above, PE films do not readily biodegrade or even are non-degradable [4-7]. After using them, the residual plastic segments of the PE films will cause detrimental soil pollution problems. The residual plastic segments will reduce soil permeability, impede crop roots from absorbing water and nutrients, and reduce the yield [8-12]. In addition, due to the lack of efficient recovery of residual PE films, according to the statistics, the ratio of the coverage of residual PE films ranges from 60 to 349 kg•ha^−1^ in the northern part of China [13], which threatens the food security and soil quality [14]. Furthermore, the release of plastic fragments from the residual PE films in the fields has been recognized as a primary source of plastic accumulation in the sea [15]. Hence, the “white revolution” which was brought from the PE films has reversed to “white pollution” for modern agriculture [16].

Hereinabove statements, many efforts have been made to synthesis various types of degradable [mulching](javascript:;) [film](javascript:;)s to try to supersede PE films to lessen environmental pollutions [17]. Biodegradable plastic [mulching](javascript:;) [film](javascript:;)s include synthetic biodegradable plastics and natural polymer blend biodegradable plastics. The synthetic biodegradable plastics are prepared by polycaprolactone (PCL) [18], polylactic acid (PLA) [19], poly adipic acid/butylene terephthalate (PBAT) [20] The natural polymer blend biodegradable plastics are synthesized by blending starch, chitin, cellulose and chitosan with PE, polyvinyl alcohol (PVA), polyester, etc. [21-24]. Though the performance of biodegradable plastic [mulching](javascript:;) [film](javascript:;)s is close to the PE films, these materials are mostly chemically synthesized, and the effects on the soil after being degraded should be further verified. Straw mulches were once suggested as an alternative to PE films because straw mulches can inhibit weeds effectively, can be degraded into organic substances without recovery, and have almost no adverse effects on soil [25, 26]. But plant fiber mulches have lower insulation and mechanical qualities than PE films, and also deteriorate swiftly into pieces and be blown away after being exposed to rain or wind within weeks [27-29].

In agriculture, spraying is a method of producing an artificial coating on the surface of plants to aid in the management of insects and diseases, fertilization, and prevention of transpiration. Sprayable polymer solutions such as oils, waxes, silicones, and a range of polymers for soil coating are widely utilized. In mulching films, spraying a continuous protective layer of biopolymer-based compositions on the soil is a new technique. The significance, possible advantages, and future obstacles of producing a cost-effective biodegradable sprayable film used in agriculture were emphasized. [30].

Cow dung, as the waste of animal husbandry, is another primary pollution in rural areas, and it has become an urgent problem to be solved. However, cow dung is not only a kind of pollution source, it is also a kind of recyclable resource at the same time, which attracts broad attention. Nowadays, it can be seen in widespread including farmland manure, clean energy [31, 32], sewage treatment [33, 34], composite materials [35, 36], and even in the preparation of the battery [37]. In addition, the soil covered with cow dung, because of the biological fermentation, exhibits more microorganism species, which improve soil fertility as well as soil properties [38-43].

Zhangye City (locates in Gansu Province, the northwest of China) is with a typical temperate continental climate, which is mainly manifested in significant changes in annual and daily temperature. It is cold in winter and hot in summer, and with less precipitation throughout the year. Meanwhile, Zhangye City is one of the largest corn seed production bases in China, and animal husbandry is another booming enterprise, which has almost 600,000 heads of castles. While benefiting located economic income, its geographical location determines that a large amount of PE films must be used before corn sowing. At the same time, animal husbandry also brings about the waste disposal problem. Thus, rational development and utilization of waste to change it into treasure and solve the “white pollution” brought from the PE films, are of significance.

To alleviate the hazards of PE films and make full use of the advantage of cow dung, here, a kind of liquid mulching film prepared by cow dung extracts was studied. Through three-year field experiments, the CDLM had thermal insulation that can increase the soil temperature by 1~3°C during the seed development period. It began to degrade about 60 days and degraded entirely in 100 days, covering the whole vegetative stage of the silage corn growth. In addition, after being degraded gradually and buried in soil, the soil organic matters covered by CDLM increased by nearly 0.3% per year, which improved the soil quality. As a result, the mean silage corn yield under CDLM was 91532 kg•ha^-1^, 17.2% and 6.2% greater than that under CK and PE films. The mean WUE of the silage corn under CDLM, PE films and CK were 200.79 kg•ha^−1^•mm^−1^, 189.08 kg•ha^−1^•mm^−1^, and 171.40 kg•ha^−1^•mm^−1^.

2. **Methods and materials**
   2.1 **Site description**

The three-year (2018-2020) field experiment was conducted in the planting and breeding base of Huarui Agricultural Company, Eco-industrial Park, Minle County, Zhangye City, Gansu Province, China (**Fig. 1**). Each experimental plot was 0.6 ha (**Fig. 1c**). The regional groundwater table remained at a depth of 8 m. The local soil was sandy with a pH of 8.38, with a mean 14.5% field water holding capacity. In the surface soil (0–20 cm depth), total N, P, and available K was 0.034g·kg^−1^, 0. 03g·kg^−1^, and 0.15g·kg^−1^, respectively. The site has been practised mulched drip irrigation since 2015. The mean soil bulk density of the topsoil (0–40 cm depth) was 1.488 g·cm^−3^.

This region has an arid continental climate. The mean annual sunshine duration is 3000 h, and the frost-free period lasts 140 days a year. The accumulated temperatures above 10 °C and 15 °C were 3169 °C and 2595°C in 2018, 3138.4 °C and 2538.4°C in 2019, and 3176.2 °C and 2584.1°C in 2020, respectively. As shown in **Fig. 2**, the precipitations and mean daily temperatures of the experimental sites during the cropping season (April to September) were 242.2 mm and 17.4 °C in 2018, 244.1 mm and 17.5°C in 2019, and 222.3 mm and 17.5°C in 2020, respectively.

2.2 **Preparation and spraying of CDLM on the plots**

The preparation process and spraying CDLM on the plots are shown in **Fig. 3**. As the preparation process shown in **Fig. 3a**, the first step was to flush the cow dung in the enclosure into the settling pond, and get the dung liquid with the sand content less than 2% through multi-stage sand settling and the impurity-removing treatments. The second step was to separate the liquid from the cow dung to obtain the cow dung with a moisture content about 70%. The separated cow dung was fermented by anaerobic and aerobic fermentation to reduce the Chemical Oxygen Demand (COD), nitrogen, and faecal coliforms on supernatant to 2500.4±500 mg•L^-1^, 550.1±75 mg•L^-1^, and ~ 358 L^-1^, respectively, for grinding pulp. The third step was to add wheat straw about 10wt.% to the treated cow dung, and to make a slurry containing dry matter about 40wt.% using faecal water.

2.3 **The design of field experiments**

The experiment was prepared by a comparison method designed in a large area with three different treatments, i.e., mulching with PE films, mulching with CDLM films, and CK. Each planting plot was 0.6 ha, and the crop planted was silage corn. The narrow/wide row alternation for silage corn was adopted. The plant spacing was 20 cm, the narrow row spacing was 25 cm, and the wide row spacing was 75 cm, as shown in **Fig. 4**. We sowed 10000 holes per 0.1 ha, preserving 9000 plants to guarantee the emergence rate. Drip irrigation under film was used in theexperiment. The flow of drip irrigation was 3 L•h^-1^, and the whole irrigation amount was 3750 m^3^•ha^-1^. The fertilizers were applied via drip irrigation laterals, and the details of the fertilizers used per 0.1 ha are shown in **Table 1**.

The drip irrigation laterals with the type of Single wing labyrinth (Tianye Co. Ltd., Xinjiang, China) were employed. The external diameter and wall thickness are 16 mm and 0.3 mm, respectively. Interval spacing between two emitters is 30 cm. A water pump was installed to exert pressure on the water supply system, and the pressure gauges were connected to the front of the pipes to monitor water pressure inside the pipe. Sluice valves were used to adjust water pressure at 0.06 MPa to maintain the flow rate of approximately 3 L•h^−1^.

To identify the stages of crop development, a standardized corn developmental staging system was used (Hanway, 1966). The date was recorded when more than 50% of the plants in each plot reached the vegetative stage (VS) and reproductive stage (RS). The VS stage includes sowing time (ST), germination and emergence (VE), V3, V6, V9, V12, V15, and VT. The RS stage includes R1 (Silking), R2 (Blister, 10~14 days after Silking), R3 (Milk, 18~22 days after Silking), R4 (Dough, 24-28 days after Silking), R5 (Dent, 35-42 days after Silking), R6 (Physiological maturity, 55-65 days after Silking). The collected periods of silage corn growth in the experimental base from 2018 to 2020 are shown in **Table 2**.

2.4 **Sampling measurements**
    2.4.1 **The degradation of** **mulching films**

To observe the degradation of mulching films, the pieces of CDLM and PE films with a size of 20cm$\times$20cm were collected randomly during the ST, VE, V6, V9, and V12 stages of the silage corn growth in 2020 and the image observation method was used.

2.4.2 **The measurement of soil temperature**

Soil temperature was measured at a depth of 10 cm in each plot using Sansheng Smart Agriculture Platform (Zhangjiakou Sansheng Smart Agriculture Technology Co, Ltd, Hebei, China). The soil temperatures were recorded at every 1 h within 24 h at a certain day in the critical stages of ST, VE, V6, V9 and V12.

**2.4.3 The measurement of soil organic matters**

Soil organic matters were measured after the annual harvest, and the samples were obtained from the plots with three different treatments, i.e., mulching with PE films, mulching with CDLM films, and CK. The test was carried out by the standards of GB 9834-88, and the mean values were determined for analysis.

**2.4.4 The measurements of the yield of silage corn and WUE**

The silage corn yield was determined by mechanized harvesting of each test plot and converted to yield per unit area (kg•ha^-1^). The WUE (kg•ha^-1^•mm^-1^) was calculated as the ratio between the annual silage corn yield and the total evapotranspiration (ET) over the growing season in each year [44]. Given the limited influence of groundwater on soil water, no surface runoff and no deep percolation at the experiment site, ET was be calculated as:

$$ET=P+I+\Delta SWS$$

where, *P* is effective rainfall (mm), *I* is irrigation (mm), and *∆SWS* (mm) is the difference in soil water storage in the depth of soil about 0-40 cm between sowing and harvest.

**2.4.5 *Statistical analysis***

The data processing software Origin2020 was used, and the differences were considered statistically significant when p<0.05.

3. **Results and discussion**
   3.1 **The degradation of mulching films**

The observation of the degradation of mulching films was from April 26, 2020, which was the day of seeding, to August 5, 2020. **Fig. 5** shows the degradations of CDLM and PE films at VS of ST, VE, V6, V9 and V12 stages in 2020. As can be seen from **Fig. 5**, the change of CDLM was more noticeable compared with PE films. After spraying CDLM for 4 days, on April 30 (at the ST stage), the moisture in the CDLM evaporated rapidly, and the surface of CDLM hardened and became a whole block gradually. The back of CDLM completely adhered to the soil, and the color of the surface of CDLM was similar to cow dung, as illustrated in **Fig. 5a**, **5b**. After 7 days, on May 3 (at the VE stage), as the CDLM hardened further, the combination of the CDLM itself became better than its initial liquid state, making it easier to collect (**Fig. 5e**, **5f**). After 27 days, on May 23 (at the V6 stage), the combination of CDLM was the best, and we can collect the complete mulching film at this time (**Fig. 5i**, **5j**). After 61 days, on June 27 (at the V9 stage), many cracks began to appear, which made it impossible to obtain a sizeable complete film (**Fig. 5m**, **5n**). After 81 days, on July 17 (at the V12 stage), the cracks have spread even further, the collection process became more complicated, and some parts of the mulch have merged with the soil (**Fig. 5q**, **5r**). After 100 days, on August 5, CDLM could no longer be collected, and CDLM was degraded entirely and fused with the soil. These results show that CDLM begins to degrade after 60 days, and it completely degrades after 100 days. In comparison, the results show that PE films remain unchanged during the whole observation, and the residual mulching films left after the harvest should be recovered to minimize soil contamination. In contrast, the CDLM can avoid this extra cost.

The effects of degradation of CDLM on the silage corn growth were also studied. It showed that, when water evaporated from the CDLM at the ST stage, CDLM hardened rapidly, the back of the side wholly adhered to the soil, and formed a covering layer. Because the additional soil was connected to the back of the CDLM during the collection of CDLM at the VE stage, the CDLM became more securely bonded to the soil from the ST stage to the VE stage. So the CDLM could provide heat preservation to the soil and effectively fix soil to prevent erosion from wind and rain, and this effect was carried over into the V6 stage. As a comparison, at the same scene, although PE films had a more significant heat preservation function than CDLM, it was quickly destroyed by wind if relevant precautions were not taken. At the VE stage, the mechanical strength of CDLM was lower than that of PE films, so seedlings could immediately break through the CDLM, and the emergence rate was guaranteed. However, the PE films need manual treatments to release seedlings, otherwise, the seedlings will die. During the V9 stage, CDLM gradually degraded to fragments, allowing more air and water into the soil. However, PE films prevented the entry of air and water into the soil (Wang et al., 2020). There was no change in the V12 stage, and CDLM were fused sufficiently in soil after the V14 stage. From the above, these findings indicate that the degradation of CDLM is more apparent, and much better than that of PE films. The progressive decrease of CDLM during crop growth establishes a solid basis for the increase of the yield.

3.2 **The measurement of soil temperature**

The air temperature and soil temperatures of plots covered by CDLM, PE films, and CK at a depth of 10 cm were recorded at every 1 h within 24 h on a certain day in the critical stages of ST, VE, V9, and V12 in 2018, 2019, and 2020, as illustrated in **Fig. 6**.

At the initial stage of planting (ST stage), in 2018, 2019, and 2020 respectively, as shown in **Fig. 6a**, **6i**, under normal weather circumstances (on April 25, 2018 and April 25, 2020), there was little difference in soil temperatures among CDLM, PE films, and CK groups on the morning. The soil temperature under PE films became higher at noon. The PE films had a maximum temperature of 5°C higher than CK at the same time interval throughout the day, while CDLM had a temperature of 1~3°C higher than CK. At 11 p.m., there was little difference either. As illustrated in **Fig. 6e**, when there was enough sunshine (on April 23, 2019), the maximum temperature (about 30°C) under PE films was reached at 1 p.m. Compared the soil temperature covered by PE films with the air temperature simultaneously, the soil temperatures were consistently higher than the air temperature, even at midnight. The soil temperature still remained close to 20°C at 11 p.m., which manifests evident thermal insulation of PE films. The capability for thermal insulation can be ranked as PE>CDLM>CK after three-year observations. Because of the primary function of mulching films for keeping soil temperature and water to promote the crops germinate in advance, the seeds sown under PE films sprouted 3-6 days earlier than CDLM, whereas seeds sown under CDLM germinated 1-2 days earlier than CK at the same period.

**Fig. 6b**, **6f**, **6j** show the variations of soil temperatures among PE films, CDLM, and CK groups during the VE stage. Under normal weather circumstances (on May 1, 2018), the change of soil temperatures in this stage was almost identical to the preceding stage. On a rainy day (on April 30, 2019), the soil temperatures under CDLM and CK were below 10°C and the changes over time were inapparent. The soil temperature under PE films was not high either, but showed a slightly better thermal insulation. As **Fig. 6j** indicates, in direct sunshine (on May 2, 2020), the soil temperature under PE films was 8°C higher than under the CDLM and CK. During this stage, as the environment gradually warms, the soil temperature rises, so the heat preservation of PE films is secondary. The excessive temperature will prevent the seedling development and even scorch seedlings, affecting the crop development instead. As a result, if seedlings are not released by manual treatments, the seedlings will be scorched, reducing the emergence rate. However, the color of CDLM in this period is darker and easier to absorb sunlight, so the soil temperature under CDLM is 1~2°C higher than that of the CK group, which will not cause damages to seedlings.

After entering the V9 stage, the air temperature continues to rise, at which time the role of mulching films is over. **Fig. 6c**, **6g**, **6k** show the soil temperatures under CDLM and CK tended to be similar. It is because CDLM begins to degrade, and the integration of the CDLM with soil deepen at this stage. However, from the perspective of degradation, there is no evident change in PE films. As the air temperature rises, the soil temperature under the PE films is higher than CDLM and CK, thus increasing the evaporation of soil water. On the other hand, due to the difference in temperatures between day and night, the surface of PE films is easy to cause water accumulation, which also harms crop growth.

In the V12 stage, as shown in **Fig. 6d**, **6h**, **6l**, the soil temperature under CDLM was nearly identical to that of CK. It is because that the CDLM has been wholly degraded at this stage, and the crop growth is completed. PE films shaded by the crop are no longer directly exposed to sunlight, the soil temperature under the PE films is close to that of under the CDLM and CK, and occasionally varies with changes in air temperature.

According to the three-year observations, the thermal insulation of PE films is superior to that of CDLM. However, the soil temperature under PE films changes rapidly sometimes, which is not conducive to crop growth. In the VE stage, the high temperature even scorches the seedlings and affects the emergence rate. In the V9 stage, the evaporation of soil water and the water accumulation on the surface of PE films also harm crop growth. The thermal insulation of CDLM is better than the CK group, and the CDLM can retain heat, enhance the buildup of soil temperature, and aid the seed germination of silage corn.

3.3 **The contents of soil organic matters**

The contents of soil organic matters under CDLM, PE films and CK after the harvest of silage corn in 2018, 2019 and 2020 were detected and shown in **Fig. 7**. In 2018, the content of soil organic matters was 1.01% inthe CDLM, 0.66% in the CK group, and 0.72% in the PE films. In 2019, the content of soil organic matters in the CDLM group was 1.27%, 0.74% in the CK group, and 0.75% in the PE films group. In 2020, the content of soil organic matters in the CDLM group was 1.51%, 0. 69% in the CK group, and 0.78% in the PE films group. The content of soil organic matters in the CDLM group increased by nearly 0.3% per year.

The quality of local soil tested is mainly sandy soil because the geographical condition is the Gobi Desert, and the content of soil organic matters is deficient, so the crops will not grow unless the nutrients in the soil are improved. Cow dung can significantly increase the content of soil organic matters, increase nutrient contents of crops, help crops absorb other nutrient elements, and improve soil physical properties, especially for land with extreme nutrient deficiency (Lovell and Jarvis, 1996). After CDLM degradation, it can effectively manifest the above effects, and these changes of the soil are not brought about from the PE films.

3.4 **Silage corn yield and water use efficiency**

The silage corn yields and WUE under CDLM, PE films and CK after the harvest in 2018, 2019 and 2020 were detected and shown in **Table 3** and **Fig. 8**. The mean silage corn yields under CDLM, PE films and CK over the three years were 91532, 86191, and 78133 kg•ha^−1^, respectively. The three-year experiments showed that the yield of the plot under CDLM was 17.2% higher than that of the CK group, and about 6.2% higher than that of the PE films. The mean WUE of the silage corn under CDLM was 200.79 kg•ha^−1^•mm^−1^, which is higher than PE films (189.08 kg•ha^−1^•mm^−1^) and increases more obviously than CK (171.40 kg•ha^−1^•mm^−1^).

Film mulching techniques had significantly improved the silage corn yield with respect to no mulching [45, 46]. The CDLM can provide favorable conditions for crop emergence at the early growth stage, warm and wet the soil, and increase the content of soil organic matters after degrading. These effects on soil amelioration are noticeable after long-term use and manifested by the high improvements in crop growth and crop yield.

4. **Conclusions**

The CDLM can lessen the soil pollution caused by non-degradable PE films and rational develop and use animal husbandry waste. The results showed that CDLM has better degradability than PE. After degradation, the content of soil organic matters was increased evidently, and the effect on soil amelioration was noticeable after long-term use. And the CDLM had an effect on thermal insulation, which promoted crop germination. As a result, higher silage corn yields and WUE were obtained. Therefore, the CDLM as a substitute for PE films and has the potential for solving the problems of waste disposal and “white pollution”.

**Acknowledgements**

Lu Li acknowledges the supports by the Gansu Province Natural Science Foundation for the Youth (20JR10RA554) and GAU-KYQD-2019-16.

**Competing interests**

The authors declare no competing interests.

**REFERENCES**

1.	Li, X., J. Gong, and X. Wei, In-situ rainwater harvesting and gravel mulch combination for corn production in the dry semi-arid region of China. Journal of Arid Environments, 2000. 46(4): p. 371-382.

2.	Kasirajan, S. and M. Ngouajio, Polyethylene and biodegradable mulches for agricultural applications: a review. Agronomy for Sustainable Development, 2012. 32(2): p. 501-529.

3.	Qin, S., et al., Effect of ridge–furrow and plastic-mulching planting patterns on yield formation and water movement of potato in a semi-arid area. Agricultural Water Management, 2014. 131: p. 87-94.

4.	Hou, L., et al., Biodegradability and ecological impacts of polyethylene-based mulching film at agricultural environment. J Hazard Mater, 2019. 378: p. 120774.

5.	Zhao, Y., et al., Simulation of soil water and heat flow in ridge cultivation with plastic film mulching system on the Chinese Loess Plateau. Agricultural Water Management, 2018. 202: p. 99-112.

6.	Ma, D., et al., Impacts of plastic film mulching on crop yields, soil water, nitrate, and organic carbon in Northwestern China: A meta-analysis. Agric Water Manag, 2018. 202: p. 166-173.

7.	Lin, W., et al., Influence of plastic film mulch on maize water use efficiency in the Loess Plateau of China. Agricultural Water Management, 2019. 224.

8.	He, H., et al., Distribution characteristics of residual film over a cotton field under long-term film mulching and drip irrigation in an oasis agroecosystem. Soil and Tillage Research, 2018. 180: p. 194-203.

9.	Xie, H., et al., Influence of Residual Plastic Film on Soil Structure Crop Growth and Developm ent in Fields. Journal of Agro-Environm ent Science, 2007. 26: p. 153-156.

10.	Ammala, A., et al., An overview of degradable and biodegradable polyolefins. Progress in Polymer Science, 2011. 36(8): p. 1015-1049.

11.	Liu, M., Z. Huang, and Y. Yang, A Study on Status and developmental Trend of biodegradable Plastic Film. Chinese Agricultural Science Bulletin, 2008. 24(9): p. 439-443.

12.	Liu, X.S., B. Loudermilk, and T. Simpson, Introduction to Sample Size Choice for Confidence Intervals Based ontStatistics. Measurement in Physical Education and Exercise Science, 2014. 18(2): p. 91-100.

13.	Dong, H., et al., Effects of plastic film residue on cotton yield and soil physical and chemical properties in Xinjiang. Journal of Agro-Environment Science, 2013. 29(8): p. 91-99.

14.	Zhou, K., et al., Ultrathin cellulose nanosheet membranes for superfast separation of oil-in-water nanoemulsions. Nanoscale, 2014. 6(17): p. 10363-10369.

15.	Sintim, H.Y. and M. Flury, Is Biodegradable Plastic Mulch the Solution to Agriculture's Plastic Problem? Environ Sci Technol, 2017. 51(3): p. 1068-1069.

16.	Kyrikou, I. and D. Briassoulis, Biodegradation of Agricultural Plastic Films: A Critical Review. Journal of Polymers and the Environment, 2007. 15(2): p. 125-150.

17.	Kader, M.A., et al., Recent advances in mulching materials and methods for modifying soil environment. Soil & Tillage Research, 2017. 168: p. 155-166.

18.	Fields, R., F. Rodriguez, and R. Finn, Microbial degradation of polyesters: polycaprolactone degraded by P. pullulans. Journal of Applied Polymer Science, 1974. 18(12): p. 3571-3579.

19.	Rocca-Smith, J.R., et al., Beyond Biodegradability of Poly(lactic acid): Physical and Chemical Stability in Humid Environments. Acs Sustainable Chemistry & Engineering, 2017. 5(3): p. 2751-2762.

20.	Rocha, D.B., et al., A new approach for flexible PBAT/PLA/CaCO3films into agriculture. Journal of Applied Polymer Science, 2018. 135(35).

21.	Zatorre, R.J., R.D. Fields, and H. Johansen-Berg, Plasticity in gray and white: neuroimaging changes in brain structure during learning. Nature neuroscience, 2012. 15(4): p. 528-536.

22.	Laxmeshwar, S.S., et al., Preparation and properties of biodegradable film composites using modified cellulose fibre-reinforced with PVA. International Scholarly Research Notices, 2012. 2012.

23.	Tan, B.K., et al., Biodegradable Mulches Based on Poly(vinyl Alcohol), Kenaf Fiber, and Urea. Bioresources, 2015. 10(3): p. 5532-5543.

24.	Liu, D.C. and F.Y. Gao, Multi-functional Characteristics of Novel Biodegradable Mulching Films from Citric Acid Fermentation Wastes. Waste and Biomass Valorization, 2018. 9(8): p. 1379-1387.

25.	Haapala, T., et al., Effects of different paper mulches on soil temperature and yield of cucumber (Cucumis sativus L.) in the temperate zone. Agricultural and Food Science, 2015. 24(1): p. 52-58.

26.	Li, A., et al., Research progress on preparation and field application of paper mulch. Environmental Technology & Innovation, 2021. 24.

27.	Sun, E., et al., Biological fermentation pretreatment accelerated the depolymerization of straw fiber and its mechanical properties as raw material for mulch film. Journal of Cleaner Production, 2021. 284.

28.	Claramunt, J., et al., Mechanical characterization of blends containing recycled paper pulp and other lignocellulosic materials to develop hydromulches for weed control. Biosystems Engineering, 2020. 191: p. 35-47.

29.	Song, X., X. Wang, and K. Kito, Effects of Heating Temperature on the Properties of Bio-Board Manufactured by Using Soybean Straw. Materials (Basel), 2020. 13(3).

30.	Adhikari, R., et al., Preformed and sprayable polymeric mulch film to improve agricultural water use efficiency. Agricultural Water Management, 2016. 169: p. 1-13.

31.	Abdeshahian, P., et al., Potential of biogas production from farm animal waste in Malaysia. Renewable and Sustainable Energy Reviews, 2016. 60: p. 714-723.

32.	Alemayehu, G., Co-digestion of municipal organic wastes with night soil and cow dung for biogas production: A Review. African Journal of Biotechnology, 2016. 15(2): p. 32-44.

33.	Lee, N., et al., Conversion of cattle manure into functional material to remove selenate from wastewater. Chemosphere, 2021. 278: p. 130398.

34.	Dias, J.M., et al., Waste materials for activated carbon preparation and its use in aqueous-phase treatment: A review. Journal of Environmental Management, 2007. 85(4): p. 833-846.

35.	Vedrtnam, A., Fabrication and characterization of cow dung- Polyvinyl Alcohol composite film. Composites Communications, 2018. 8: p. 31-35.

36.	Saikishore, T., M.P.P. Rao, and D.M.C.S. Reddy, Synthesis & Investigation of the Mechanical behaviour of Luffa Groundnut Shell, Chicken Feather and Cowdung Fibers Reinforced Epoxy Composites. International Journal for Scientific Research & Development, 2017. 5(4): p. 42-46.

37.	Rajabathar, J.R., et al., Flexible Type Symmetric Supercapacitor Electrode Fabrication Using Phosphoric Acid-Activated Carbon Nanomaterials Derived from Cow Dung for Renewable Energy Applications. ACS Omega, 2020. 5(25): p. 15028-15038.

38.	Zhang, L. and X. Sun, Using cow dung and spent coffee grounds to enhance the two-stage co-composting of green waste. Bioresour Technol, 2017. 245(Pt A): p. 152-161.

39.	Ali, U., et al., A review on vermicomposting of organic wastes. Environmental Progress & Sustainable Energy, 2015. 34(4): p. 1050-1062.

40.	Yadav, A., R. Gupta, and V.K. Garg, Organic manure production from cow dung and biogas plant slurry by vermicomposting under field conditions. 2, 2013. 21: p. 1-7.

41.	Nattudurai, G., et al., Vermicomposting of coirpith with cowdung by Eudrilus eugeniae Kinberg and its efficacy on the growth of Cyamopsis tetragonaloba (L) Taub. Journal of the Saudi Society of Agricultural Sciences, 2014. 13(1): p. 23-27.

42.	Syranidou, E., et al., Biodegradation of mixture of plastic films by tailored marine consortia. J Hazard Mater, 2019. 375: p. 33-42.

43.	Yuan, J., et al., Microbial degradation and other environmental aspects of microplastics/plastics. Sci Total Environ, 2020. 715: p. 136968.

44.	Ghulam Hussain *, A.A.A.-J., Effect of irrigation and nitrogen on water-use efficiency of wheat in Saudi Arabia. Agricultural Water Management, 1995. 27: p. 143-153.

45.	Yin, W., et al., Integrated double mulching practices optimizes soil temperature and improves soil water utilization in arid environments. Int J Biometeorol, 2016. 60(9): p. 1423-37.

46.	Fan, Y., et al., Plastic mulch decreases available energy and evapotranspiration and improves yield and water use efficiency in an irrigated maize cropland. Agricultural Water Management, 2017. 179: p. 122-131.

**Figure Captions**

**Fig. 1.** The location of Gansu Province in China (a); The location of Huarui agricultural company, Eco-industrial park, Minle County, Zhangye City in Gansu Province (b); The location of experimental plots in Zhangye City (c).

**Fig. 2.** The precipitations (indicated by the bars) and mean daily temperatures (indicated by the curves) of the experimental sites in the growing season of the silage corn in 2018, 2019, 2020.

**Fig. 3.** The process of preparation of CDLM (a); Spraying of CDLM on the plots (b); The plots after being sprayed (c).

**Fig. 4.** The narrow/wide row alternation for silage corn in experimental plots.

**Fig. 5.** The degradations of CDLM and PE films at VS of ST, VE, V6, V9 and V12 stages in 2020.

**Fig. 6.** The air temperature and soil temperatures of plots covered by CDLM, PE films, and CK recorded at every 1 h within 24 h at a certain day in the critical stages of ST, VE, V9 and V12 in 2018, 2019 and 2020.

**Fig. 7.** The contents of soil organic matters under CDLM, PE films and CK after the harvest in 2018, 2019 and 2020. Different superscripted letters (a, b, and c) indicate significant differences amongst the treatments in a year (p<0.05).

**Fig. 8.** The silage corn yields under CDLM, PE films and CK in 2018, 2019 and 2020. Different superscripted letters (a, b, and c) indicate significant differences amongst the treatments in a year (p<0.05).

**Tables and Figures**

**Table 1** The details of the fertilizers used per 0.1 ha.

| Growth Stage | Fertilization rule (kg) | | |
| --- | --- | --- | --- |
|  | CO(NH_2_) _2_ | CaP_2_H_4_O_8_ | KCl |
| V3 | 8.33 | 1.67 | 3.33 |
| V6 | 16.67 | 3.33 | 3.33 |
| V10 | 16.67 | 5 | 13.33 |
| V14 | 16.67 | 3.33 | 5 |
| V17 | 16.67 | 5 | 8.33 |
| VT | 16.67 | 5 | 6.67 |
| R1 | 8.33 | 1.67 | 6.67 |
| **Total** | 100 | 25 | 46.67 |

**Table 2** The collected periods of silage corn growth in the experimental base from 2018 to 2020.

|  | ST | VE | V6 | V9 | V12 | R1 | R6 |
| --- | --- | --- | --- | --- | --- | --- | --- |
| 2018 | 24.Apr | 1.May | 19.May | 24.Jun | 14.Jul | 31.Jul | 24.Sept |
| 2019 | 23.Apr | 30.Apr | 18.May | 23.Jun | 13Jul | 30. Jul | 23.Sept |
| 2020 | 25.Apr | 2.May | 20.May | 25.Jun | 15.Jul | 1. Aug | 25.Sept |

**Table 3** The silage corn yields and water use efficiency (WUE) under CDLM, PE films and CK in 2018, 2019, and 2020. Different letters (a, b, and c) within a column indicate significant differences amongst the treatments in a year (p<0.05).

| Growing season | Treatment | Yield (kg•ha^−1^) | ET (mm) | WUE(kg•ha^−1^•mm^−1^) |
| --- | --- | --- | --- | --- |
| 2018 | CDLM | 91381.08a | 455.62 | 200.56a |
|  | PE | 86036.32b |  | 188.83b |
|  | CK | 78093.44c |  | 171.40c |
| 2019 | CDLM | 91591.23a | 453.35 | 202.03a |
|  | PE | 86576.65b |  | 190.97b |
|  | CK | 77777.84c |  | 171.56c |
| 2020 | CDLM | 91621.35a | 458.58 | 199.79a |
|  | PE | 85960.52b |  | 187.45b |
|  | CK | 78528.49c |  | 171.24c |

**Fig. 1.** The location of Gansu Province in China (a); The location of Huarui agricultural company, Eco-industrial park, Minle County, Zhangye City in Gansu Province (b); The location of experimental plots in Zhangye City (c).

**Fig. 2.** The precipitations (indicated by the bars) and mean daily temperatures (indicated by the curves) of the experimental sites in the growing season of the silage corn in 2018, 2019, 2020.

**Fig. 3.** The process of preparation of CDLM (a); Spraying of CDLM on the plots (b); The plots after being sprayed (c).

**Fig. 4.** The narrow/wide row alternation for silage corn in experimental plots.

**Fig. 5.** The degradations of CDLM and PE films at VS of ST, VE, V6, V9 and V12 stages in 2020.

**Fig. 6.** The air temperature and soil temperatures of plots covered by CDLM, PE films, and CK recorded at every 1 h within 24 h at a certain day in the critical stages of ST, VE, V9 and V12 in 2018, 2019 and 2020.

**Fig. 7.** The contents of soil organic matters under CDLM, PE films and CK after the harvest in 2018, 2019 and 2020. Different superscripted letters (a, b, and c) indicate significant differences amongst the treatments in a year (p<0.05).

**Fig. 8.** The silage corn yields under CDLM, PE films and CK in 2018, 2019 and 2020. Different superscripted letters (a, b, and c) indicate significant differences amongst the treatments in a year (p<0.05).
