# Supplementary material for: Substitute for polyethylene (PE) films: A novel cow dung-based liquid mulch on silage cornfields
Source: PLoS One. 2022 Jul 13;17(7):e0271273. doi: 10.1371/journal.pone.0271273 (PMC9278788; doi:10.1371/journal.pone.0271273)
Supplement: Attachment — Submitted filename: Response to Reviewers.docx [file pone.0271273.s009.docx]

Dear Raghvendra Bohara,

We would like to thank you for your consideration of our manuscript and thank Reviewers for the detailed and constructive comments. Please find our point-by-point responses in the following pages and changes in the manuscript highlighted in blue. We hope you and the reviewer will find the responses and changes satisfactory.

With best regards,

Wuyun Zhao

PLOS ONE (PONE-D-21-39586)

**Substitute for polyethylene (PE) films: a novel cow dung-based** **liquid mulch on** **silage cornfields**

Xiangjun Yang^a,b┼^, Lu Lia^┼^, Wuyun Zhao^a,*^, Xuan Li^c^, Yongsong Mu^d^, Maohan Chen^b^, Xiaoqiang Wu^b^

**Response for the Journal Requirements:**

**Dear Editor:**

Thank you for your useful comments and suggestions on our manuscript. We have modified the manuscript accordingly. We present here the detailed response to the journal requirements on our work point by point as below:

**1. Please ensure that your manuscript meets PLOS ONE's style requirements, including those for file naming. The PLOS ONE style templates can be found at** [**https://journals.plos.org/plosone/s/file?id=wjVg/PLOSOne_formatting_sample_main_body.pdf**](https://journals.plos.org/plosone/s/file?id=wjVg/PLOSOne_formatting_sample_main_body.pdf) **and** [**https://journals.plos.org/plosone/s/file? id=ba62/PLOSOne_ formatting**](https://journals.plos.org/plosone/s/file?%20id=ba62/PLOSOne_%20formatting) **sample title _authors_affiliations.pdf**

**Reply to Editor:**

We have revised and checked the style of manuscript and now it meets the style templates of PLOS ONE.

**2. PLOS requires an ORCID iD for the corresponding author in Editorial Manager on papers submitted after December 6th, 2016. Please ensure that you have an ORCID iD and that it is validated in Editorial Manager. To do this, go to ‘Update my Information’ (in the upper left-hand corner of the main menu), and click on the Fetch/Validate link next to the ORCID field. This will take you to the ORCID site and allow you to create a new iD or authenticate a pre-existing iD in Editorial Manager. Please see the following video for instructions on linking an ORCID iD to your Editorial Manager account:** [**https://www.youtube.com/watch**](https://www.youtube.com/watch)**? v=_xcclfuvtxQ**

**Reply to Editor:**

We have an ORCID iD.

Xiangjun Yang: <https://orcid.org/0000-0002-2885-0117>

Wuyun Zhao: <https://orcid.org/0000-0002-6012-3710>

Lu li: https://orcid.org/0000-0003-2766-6219

**3. We note that Figure 1 in your submission contain [map/satellite] images which may be copyrighted. All PLOS content is published under the Creative Commons Attribution License (CC BY 4.0), which means that the manuscript, images, and Supporting Information files will be freely available online, and any third party is permitted to access, download, copy, distribute, and use these materials in any way, even commercially, with proper attribution. For these reasons, we cannot publish previously copyrighted maps or satellite images created using proprietary data, such as Google software (Google Maps, Street View, and Earth). For more information, see our copyright guidelines:** [**http://journals.plos.org/plosone/s/**](http://journals.plos.org/plosone/s/) **licenses-and-copyright. We require you to either (1) present written permission from the copyright holder to publish these figures specifically under the CC BY 4.0 license, or (2) remove the figures from your submission:**

**Reply to Editor:**

The **Figure 1** were removed in the revised manuscript.

**4. Thank you for stating the following in the Acknowledgments Section of your manuscript: “Lu Li acknowledges the supports by the Gansu Province Natural Science Foundation for the Youth (20JR10RA554) and GAU-KYQD-2019-16.” We note that you have provided additional information within the Acknowledgements Section that is not currently declared in your Funding Statement. Please note that funding information should not appear in the Acknowledgments section or other areas of your manuscript. We will only publish funding information present in the Funding Statement section of the online submission form. Please remove any funding-related text from the manuscript and let us know how you would like to update your Funding Statement. Currently, your Funding Statement reads as follows: “The funders had no role in study design, data collection and analysis, decision to publish, or preparation of the manuscript.” Please include your amended statements within your cover letter; we will change the online submission form on your behalf.**

**Reply to Editor:**

We have removed the funding-related text from the manuscript, but we would like to update our Funding Statement (Lu Li acknowledges the supports by the Gansu Province Natural Science Foundation for the Youth (20JR10RA554) and GAU-KYQD-2019-16), so we added the amended statements in our cover letter and remove it from our manuscript, so please change the online submission form on our behalf.

**5. In your Data Availability statement, you have not specified where the minimal data set underlying the results described in your manuscript can be found. PLOS defines a study's minimal data set as the underlying data used to reach the conclusions drawn in the manuscript and any additional data required to replicate the reported study findings in their entirety. All PLOS journals require that the minimal data set be made fully available. For more information about our data policy, please see http://journals.plos.org/plosone/s/data-availability. Upon re-submitting your revised manuscript, please upload your study’s minimal underlying data set as either Supporting Information files or to a stable, public repository and include the relevant URLs, DOIs, or accession numbers within your revised cover letter. For a list of acceptable repositories, please see http://journals.plos.org/plosone/s/data-availability#loc-recommended repositories. Any potentially identifying patient information must be fully anonymized. Important: If there are ethical or legal restrictions to sharing your data publicly, please explain these restrictions in detail. Please see our guidelines for more information on what we consider unacceptable restrictions to publicly sharing data: http://journals.plos.org/plosone/s/data-availability#loc-unacceptable-data-access-restrictions. Note that it is not acceptable for the authors to be the sole named individuals responsible for ensuring data access.**

**Reply to Editor:**

We upload our study’s minimal underlying data set as Supporting Information files. We added the following information in cover letter: All relevant data are within the paper and its Supporting Information files.

**Response for the Reviewer:**

**Dear Reviewer and Editor:**

Thank you for your useful comments and suggestions on our manuscript. We have modified the manuscript accordingly. We present here the detailed response to the reviewers' comments on our work point by point as below:

**1.The abstract is verbal description of the studies, it needs to be more quantitative, also the test crop, and its management is not been provided in the abstract.**

**Reply to Reviewer:**

We thank the reviewer for this helpful suggestion, which shall be followed. We re-organized and rewrote the abstract in the revised manuscript according to the annotated points, and we added the quantitative results, the test crop, and its management in the abstract in the revised version. The management of the test crop is presented in “Methods and materials” section

**Modification of Manuscript:**

**Line 25-36, Page 2:**

To prevent soil pollution caused by polyethylene (PE) films in the central region of Gansu, China, liquid mulching made from cow dung (CDLM) was trailed in silage maize fields. The degradation of CDLM and PE films, soil temperature, soil organic matter content, silage maize yield and water use efficiency (WUE) were evaluated for three years (2018-2020). The degradability of CDLM has been found to be much stronger than the one of PE films, with CDLM degrading 40-60 days after sowing and finishing around 100 days. CDLM had a lower insulating impact than PE films but a higher insulating effect than non-mulching films as the control (CK); CDLM could successfully increase soil organic matter, with a total increase of 1.01% over three years. CDLM increased silage maize yield by 6.2% compared to PE films and 17.2% compared to CK. Consequently, CDLM may be an interesting alternative to PE films for enhancing silage maize yield while decreasing soil contamination.

**2.The introduction section lacks objective/hypothesis, and has blended with some results particularly in the last paragraph, which is supposed to be a justification and have objectives/hypothesis**.

**Reply to Reviewer:**

We thank the reviewer for this helpful suggestion, which shall be followed. We carefully corrected the introduction in the revised manuscript according to the annotated points. And we delated the results in the last paragraph and emphasized the objectives/hypothesis in the revised version. 90-91

**Modification of Manuscript:**

We revised the introduction totally, as present in the revised manuscript.

**3.** **Fig. 2 the average temperature is misleading please provide the max and min temperature of the exp location.**

**Reply to Reviewer:**

We thank the reviewer for this helpful suggestion, which shall be followed. We replaced the **Fig 2** with a new figure and it was named **Fig 1**. The precipitations (indicated by the bars) and the max and min daily temperatures (indicated by the curves) of the experimental sites in the growing season of the silage corn in 2018, 2019, 2020.).

**Modification of Manuscript:**

**Line 122, Page 6:**

Fig 1. The precipitations (indicated by the bars) and the max and min daily temperatures (indicated by the curves) of the experimental sites in the growing season of the silage corn in 2018, 2019, 2020.

**4.Fig. 3, no need of provision Fig. 3b and 3c**

**Reply to Reviewer:**

We thank the reviewer for this helpful suggestion, which shall be followed. We deleted the Fig.3b and 3c. Now it presents as Fig.2 in revised manuscript.

**Modification of Manuscript:**

**Line 139, Page 7:**

Fig 2. The process of preparation and spraying of CDLM.

**5. In methodology section, no crop husbandry procedure is not provided, how much was the seed rate, when was sown, how it was sown, sowing time/methods etc.**

**Reply to Reviewer:**

We thank the reviewer for this helpful suggestion, which shall be followed. The crop husbandry procedure is provided in methodology section

**Modification of Manuscript:**

**Line 142-153, Page 8-9:**

Three treatments were experimented: (i) PE films, (ii) CDLM and (iii) CK. Green organic cropping patterns and mechanical harvesting methods were employed. The experiment had a random block design, with a plot area of 0.2 ha and three repetitions, 90kg•ha-1 of PE films and 60 tons•ha-1 of CLDM. Variety: Long silage No.1 was selected. The plant spacing was 20 cm, the narrow row spacing was 25 cm, and the wide row spacing was 75 cm, as shown in **Fig 3**. We sowed 10000 holes per 0.1 ha, preserving 9000 plants to guarantee the emergence rate. Drip irrigation under film was used in the experiment. The flow of drip irrigation was 3 L•h-1, and the whole irrigation amount was 3750 m3•ha-1. Urea (CO(NH2)2 with N content of 46%), monopotassium phosphate (KH2PO4, with P2O5 content of 52%) and KCl (with K of 57%) were applied via drip irrigation laterals. Detailed information on irrigation scheduling at different cotton growth stages is shown in **Table 1**.

**6. Table 1, the fertilization treatment is not clear, please change it to hectare base for clarity, also the calculation shows the N, P, K or the sources been used.**

**Reply to Reviewer:**

We thank the reviewer for this helpful suggestion, which shall be followed. We revised the **Table 1** as this comment.

**Modification of Manuscript:**

**Line 150-158, Page 9:**

Urea (CO(NH2)2 with N content of 46%), monopotassium phosphate (KH2PO4, with P2O5 content of 52%) and KCl (with K of 57%) were applied via drip irrigation laterals. Detailed information on irrigation scheduling at different silage corn growth stages is shown in Table 1.

**Table 1** **Detailed information on irrigation scheduling at different silage corn growth stages used per ha.**

| Growth Stage | Fertilization rule (kg) | | |
| --- | --- | --- | --- |
|  | CO(NH_2_) _2_ | CaP_2_H_4_O_8_ | KCl |
| V3 | 83.3 | 16.67 | 33.33 |
| V6 | 166.67 | 33.33 | 33.33 |
| V10 | 166.67 | 50 | 133.33 |
| V14 | 166.67 | 33.33 | 50 |
| V17 | 166.67 | 50 | 83.33 |
| VT | 166.67 | 50 | 66.67 |

**7. Table 2 is not need as it contains no significant information**.

**Reply to Reviewer:**

We thank the reviewer for this helpful suggestion, which shall be followed. We deleted the **Table 2**.

**8. Procedure for determination of the various parameters is not provided or lack some key information. Since the experiment was not been conducted according to any experimental design, lack of replication and randomization make the analysis impossible, then how the experimental data was analyzed, and means were compared using prob value of 5%. This section should be carefully revised.**

**Reply to Reviewer:**

We thank the reviewer for this helpful suggestion, which shall be followed. We revised the relative section as this comment.

**Modification of Manuscript:**

**Line 142-145, Page 7:**

Three treatments were experimented: (i) PE films, (ii) CDLM and (iii) CK. Green organic cropping patterns and mechanical harvesting methods were employed. The experiment had a random block design, with a plot area of 0.2 ha and three repetitions, 90kg•ha-1 of PE films and 60 tons•ha-1 of CLDM.

**Line 204-205, Page 10:**

The data processing software Origin2020 was used, and the statistical analyses were used SPSS 24.0 when p<0.05.

**9. Fig.5, what is surface ad back, not clear, also please proved the scale in days on the a-axis and add pictorial diagram after 100 days as well.**

**Reply to Reviewer:**

We thank the reviewer for this helpful suggestion, which shall be followed. We defined the surface and back of CDLM and added pictorial diagram after 100 days as shown in **Fig. 4u** and **4v** as well.

**Modification of Manuscript:**

**Line 210-214, Page 10-11:**

**Fig. 4a, 4e, 4i, 4m, 4q** and **4u** are the surface of CDLM, which was contacted with air. **Fig. 4b, 4f, 4j, 4n, 4r** and **4v** are the back of CDLM, which was contacted with soil. On April 30 (at the ST stage, four days later of CDLM spraying), the moisture in the CDLM evaporated rapidly, and the surface of CDLM hardened and became a whole block gradually.

**Line 231-233, Page 12:**

**Fig. 4.** The degradations of CDLM and PE films at VS of ST, VE, V6, V18, R2 and R4 stages in 2020.

**10. Fig 7 and 8, how these letters have been assigned, which statistical test was been used for this**.

**Reply to Reviewer:**

We thank the reviewer for this helpful suggestion, which shall be followed.

**Modification of Manuscript:**

**Line 203-205, Page 10:**

**Statistical analysis**

The data processing software Origin2020 was used, and the statistical analyses were used SPSS 24.0 when p<0.05.

**Line 331-334, Page 17:**

Fig 7. The contents of soil organic matters under CDLM, PE films and CK after the harvest in 2018, 2019 and 2020. Different superscripted letters (a, b, and c) indicate significant differences between the treatments in each year (p<0.05).

**Line 364-367, Page 19:**

**Fig 8. The silage corn yields under CDLM, PE films and CK in 2018, 2019 and 2020. Different superscripted letters (a, b, and c) indicate significant differences between the treatments in each year (p<0.05).**

**11. Table 3, no need of decimal points for the yield and WUE.**

**Reply to Reviewer:**

We thank the reviewer for this helpful suggestion, which shall be followed. We modified the **Table 3** in our revised manuscript. We delated the decimal points for the yield and WUE.

**Modification of Manuscript:**

**Line 368-370, Page 19-20:**

**Table 3 The silage corn yields and water use efficiency (WUE) under CDLM, PE films and CK in 2018, 2019, and 2020. Different letters (a, b, and c) within a column indicate significant differences between treatments in a year (p<0.05).**

| Growing season | Treatment | Yield (kg•ha^−1^) | ET (mm) | WUE (kg•ha^−1^•mm^−1^) |
| --- | --- | --- | --- | --- |
| 2018 | CDLM | 91381a | 455 | 201a |
|  | PE | 86036b |  | 189b |
|  | CK | 78093c |  | 172c |
| 2019 | CDLM | 91591a | 453 | 202a |
|  | PE | 86576b |  | 191b |
|  | CK | 77777c |  | 172c |
| 2020 | CDLM | 91621a | 458 | 200a |
|  | PE | 85960b |  | 188b |
|  | CK | 78528c |  | 171c |

**12. The results are not according the standard journal manuscript, has described only what has been provided in the table/figure. I do not see any discussion, mechanism for improvement or decline in observed parameter in response to treatment used.**

**Reply to Reviewer:**

We thank the reviewer for this helpful suggestion, which shall be followed. **Modification of Manuscript:**

**Line 234-239, Page 12-13:**

At the VE stage, the mechanical strength of CDLM was lower than that of PE films, which benefits seedlings breaking through the CDLM, thus guaranteeing emergence rate, as shown in **Fig 5a** and **5c**. In comparison, at the VE stage, the PE films need manual treatments to solve mechanized seeding caused dislocation (between membrane hole and seed hole) to release seedlings. If not, some covered seedings would die when the PE film's strength is too high to penetrate, as shown in **Fig 5b** and **5d**.

**Line 271-277, Page 15:**

Because of the primary function of mulching films for keeping soil temperature and water to promote the crops germinate in advance, the seeds sown under PE films sprouted 3-6 days earlier than CDLM, whereas seeds sown under CDLM germinated 1-2 days earlier than CK at the same period, as shown in **Table 2**.

Table 2 The seeding day and the germination day of the silage corn seeds under CDLM, PE films and CK in 2018, 2019, and 2020

|  |  | PE films | CDLM | CK |
| --- | --- | --- | --- | --- |
|  | ST | GT | GT | GT |
| 2018 | 24.Apr | 1.May | 3.May | 6.May |
| 2019 | 23.Apr | 30.Apr | 3.May | 7.May |
| 2020 | 25.Apr | 2.May | 4.May | 7.May |

**Line 326-345, Page 17-18:**

Soil organic matter plays a very important role in maintaining soil quality and improving crop yields [45]. PE films improve yields by isolating crops and enabling earlier crop development; however, studies have shown that there is a significant loss of SOM content within 1 to 3 years after mulching due to temperature-induced, accelerated biodegradation [46,47]. After crop harvest, polyethylene mulch cannot be fully recovery. The residual PE films is difficult to degradation which changes soil structure and make it susceptible to weathering, thus reducing the stability of soil structure and decreasing soil quality [46,48,49]. In comparison, residues induced by CDLM degradation, including straw fibers and cow dung, are beneficial to binding the soil generating larger soil aggregates. This extra-SOM plays an important role on macroscopic soil structure stabilization to generate organic clay and mineral complexes as a binding agent at the microscopic level [46,50].

In this work, the local soils tested were mainly sandy due to their geographical proximity to the Gobi. The liquid film based on cow dung increases the soil organic matter content, improves the soil physicochemical properties and stabilizes the soil structure. It effectively helps crops to absorb other nutrients, especially for lands that are extremely nutrient deficient. The test results showed that the use of CDLM effectively increased the organic matter content of the soil, which was twice as high after three years of continuous use as compared to unused soil, while this effect could not be observed on land with PE films.

**Line 362-373, Page 20:**

Film mulching techniques had significantly improved the silage corn yield with respect to no mulching [51,52]. Through our study, there are three reasons for the crop yield increase. First, although the CDLM covered field presents a little less soil temperature than PE films, the CDLM provides a more satisfactory condition for the initial crop growth than PE films, such as the low strength for seedling breaking and acceptable heat and water preservation effect. Second, the excellent degradation properties of CDLM promote the air injection to maintain soil the soil-air and increase the soil's organic matter, which PE films do not have. Third, the gradual degradation of CDLM increased the contacting of soil and air increasing water utilization on the crop roots when it rains, thus increasing crop yields [44,53]. At last, after three years of trials, it was shown that the characteristics of CDLM would further enhance the soil environment and finally promoting the growth of the crop and increasing the crop yield.

**13. Most of the figure/tables are not needed.**

**Reply to Reviewer:**

We thank the reviewer for this helpful suggestion, we deleted some unimportant figures and tables in our revised manuscript.

**14.Specific comments, please see** **the annotated manuscript attached.**

**Reply to Reviewer:**

We revised the manuscript as this specific comment. The detailed modifications have been shown in manuscript one by one as blue font.

**Modification of Manuscript:**

**Line 25-36, Page 2:**

To prevent soil pollution caused by polyethylene (PE) films in the central region of Gansu, China, liquid mulching made from cow dung (CDLM) was trailed in silage maize fields. The degradation of CDLM and PE films, soil temperature, soil organic matter content, silage maize yield and water use efficiency (WUE) were evaluated for three years (2018-2020). The degradability of CDLM has been found to be much stronger than the one of PE films, with CDLM degrading 40-60 days after sowing and finishing around 100 days. CDLM had a lower insulating impact than PE films but a higher insulating effect than non-mulching films as the control (CK); CDLM could successfully increase soil organic matter, with a total increase of 1.01% over three years. CDLM increased silage maize yield by 6.2% compared to PE films and 17.2% compared to CK. Consequently, CDLM may be an interesting alternative to PE films for enhancing silage maize yield while decreasing soil contamination.

**Line 40-44, Page 2:**

The mulching technique is widely used in agriculture to ensure and enhance crop yields, especially in locations with little rainfall and cold weather. The polyethylene (PE) films, which are petroleum-based products, are effective in the conservation of soil water, regulation of soil temperature, control of the weed, and improvements in water use efficiency (WUE) and yield [1–3].

**Line 47-50, Page 2-3:**

The residual plastic segments will reduce soil permeability and impede water and nutrients absorption resulting in crop yield reduction [8–10]. Moreover, due to the low recovery rate of used-PE films in north of China, the amount of residual PE films per hectare has now reached a new level of ~60-349 kg [11],

**Line 70-79, Page 3-4:**

Along with material advancements, nowadays, much attentions have been taken on the way of mulch laying. In agriculture, the liquid film is another new mulching film, which can be laid efficiently via spraying. This spray method would produce an artificial coating on the surface of plants to avoid insects, diseases and water transpiration. Several sprayable biodegradable solutions for soil mulching have been developed by utilizing natural polymers such as starch, cellulose, chitosan, alginate and glucomannan [27]. Chiellini developed a sprayable film by using waste gelatine and PVA mixes including sugar cane bagasse [28]. Adhikari recently reported a new sprayable biodegradable polymer membrane, water dispersed, which can be used in irrigated systems to prevent evaporating [29].

**Line 84, Page 4:**

composite materials [34]

**Line 93-95, Page 4:**

At the same time, animal husbandry generates waste disposal problem, with approximately 6.48×106 tons of cow dung per year [41].

**Line 97-101, Page 4:**

In this work, we produced CDLM from cow dung and then sprayed it to silage maize fields after seeding of crops. A three-year field study was conducted to investigate the degradation and insulating properties of CDLM, the effect of CDLM on the soil organic matter (SOM) content at a depth of 10 cm, and the impact of CDLM on the water use efficiency (WUE) and yield of the crop.

**Line 105, Page 5:**

(100 625 ′N, 38 375 ′E, 1673 m ASL)

**Line 109, Page 5:**

SOM 10.9 g·kg−1, pH = 8.38,

**Line 112, Page 5:**

(0–20 cm depth)

**Line 120-123, Page 6:**

Fig 1. The precipitations (indicated by the bars) and the max and min daily temperatures (indicated by the curves) of the experimental sites in the growing season of the silage corn in 2018, 2019, 2020.

**Line 132-137, Page 7:**

In the third step, the pulped cow dung was mixed with crushed wheat straw in a ratio of 3:2, and water was added to a solid-liquid ratio of 1:9. After sowing, the configured CDLM was transported by a transport vehicle (20 tons capacity) and sprayed on the field (60 tons per hectare).

Fig 2. The process of preparation and spraying of CDLM.

**Line 139-144, Page 7-8:**

Three treatments were experimented with: (i) PE films, (ii) CDLM and (iii) CK. Green organic cropping patterns and mechanical harvesting methods were employed. The experiment had a random block design, with a plot area of 0.2 ha and three repetitions, 90kg•ha-1 of PE films and 60 tons•ha-1 of CLDM. Variety: Long silage No.1 was selected. The plant spacing was 20 cm, the narrow row spacing was 25 cm, and the wide row spacing was 75 cm, as shown in **Fig 3**.

**Line 147-150, Page 8:**

Urea (CO(NH2)2 with N content of 46%), monopotassium phosphate (KH2PO4, with P2O5 content of 52%) and KCl (with K of 57%) were applied via drip irrigation laterals. Detailed information on irrigation scheduling at different cotton growth stages is shown in Table 1.

**Line 156-157, Page 8:**

**Table 1. Detailed information on irrigation scheduling at different cotton growth stages used per 0.1 ha.**

| Growth Stage | Fertilization rule (kg) | | |
| --- | --- | --- | --- |
|  | CO(NH_2_) _2_ | CaP_2_H_4_O_8_ | KCl |
| V3 | 83.3 | 16.67 | 33.33 |
| V6 | 166.67 | 33.33 | 33.33 |
| V10 | 166.67 | 50 | 133.33 |
| V14 | 166.67 | 33.33 | 50 |
| V17 | 166.67 | 50 | 83.33 |
| VT | 166.67 | 50 | 66.67 |

**Line 163-164, Page 8:**

V3 (third leaf), V6 (sixth leaf), V9 (ninth leaf), V12 (twelfth leaf), V15 (fifteenth leaf), and VT (tasseling).

**Line 181-186, Page 9-10:**

Soil samples were collected by randomly taking six cores on October 15th, 2018, October 20th, 2019, October 16th, 2020, (after silage corn harvest) from each plot (10 cm depth). The six soil cores were bulked to form a composite soil sample. The samples were then air dried, grinded and sieved through 2 mm sieves and analyzed further for the measurement of SOM.

**Line 198-199, Page 10:**

The data processing software Origin2020 was used, and the statistical analyses were used SPSS 24.0 when p<0.05.

**Line 204-208, Page 10-11:**

Fig. 4a, 4e, 4i, 4m, 4q and 4u are the surface of CDLM, which was contacted with air. Fig. 4b, 4f, 4j, 4n, 4r and 4v are the back of CDLM, which was contacted with soil. On April 30 (at the ST stage, four days later of CDLM spraying), the moisture in the CDLM evaporated rapidly, and the surface of CDLM hardened and became a whole block gradually.

**Line 234-239, Page 12-13:**

At the VE stage, the mechanical strength of CDLM was lower than that of PE films, which benefit for seedlings break through the CDLM, thus guarantee emergence rate, shown in **Fig 5a** and **5c**. In comparison, at the VE stage, the PE films need manual treatments to solve mechanized seeding caused dislocation (between membrane hole and seed hole) to release seedlings. If not, some covered seedings would dead when the PE films strength is too higher too penetrate, as shown in Fig **5b** and **5d**.

**Line 254-256, Page 13:**

Fig 5. The silage corn in experimental plots under the CDLM (a and c) and PE (b and d) films at VE stage

**Line 250, Page 13:**

outdoor temperature

**Line 271-275, Page 15:**

Because of the primary function of mulching films for keeping soil temperature and water to promote the crops germinate in advance, the seeds sown under PE films sprouted 3-6 days earlier than CDLM, whereas seeds sown under CDLM germinated 1-2 days earlier than CK at the same period, as shown in Table 2.

**Line 284--285, Page 15:**

Table 2 The seeding day and the germination day of the silage corn seeds under CDLM, PE films and CK in 2018, 2019, and 2020

|  |  | PE films | CDLM | CK |
| --- | --- | --- | --- | --- |
|  | ST | GT | GT | GT |
| 2018 | 24.Apr | 1.May | 3.May | 6.May |
| 2019 | 23.Apr | 30.Apr | 3.May | 7.May |
| 2020 | 25.Apr | 2.May | 4.May | 7.May |

**Line 331-334, Page 17:**

Fig 7. The contents of soil organic matters under CDLM, PE films and CK after the harvest in 2018, 2019 and 2020. Different superscripted letters (a, b, and c) indicate significant differences between the treatments in each year (p<0.05).

**Line 326-345, Page 17-18:**

Soil organic matter plays a very important role in maintaining soil quality and improving crop yields [45]. PE films improve yields by isolating crops and enabling earlier crop development; however, studies have shown that there is a significant loss of SOM content within 1 to 3 years after mulching due to temperature-induced, accelerated biodegradation [46,47]. After crop harvest, polyethylene mulch cannot be fully recovery. The residual PE films is difficult to degradation which changes soil structure and make it susceptible to weathering, thus reducing the stability of soil structure and decreasing soil quality [46,48,49]. In comparison, residues induced by CDLM degradation, including straw fibers and cow dung, are beneficial to binding the soil generating larger soil aggregates. This extra-SOM plays an important role on macroscopic soil structure stabilization to generate organic clay and mineral complexes as a binding agent at the microscopic level [46,50].

In this work, the local soils tested were mainly sandy due to their geographical proximity to the Gobi. The liquid film based on cow dung increases the soil organic matter content, improves the soil physicochemical properties and stabilizes the soil structure. It effectively helps crops to absorb other nutrients, especially for lands that are extremely nutrient deficient. The test results showed that the use of CDLM effectively increased the organic matter content of the soil, which was twice as high after three years of continuous use as compared to unused soil, while this effect could not be observed on land with PE films..

**Line 364--367, Page 19-20:**

Fig 8. The silage corn yields under CDLM, PE films and CK in 2018, 2019 and 2020. Different superscripted letters (a, b, and c) indicate significant differences between the treatments in each year (p<0.05).

**Line 368--370, Page 19-20:**

Table 3 The silage corn yields and water use efficiency (WUE) under CDLM, PE films and CK in 2018, 2019, and 2020. Different letters (a, b, and c) within a column indicate significant differences between treatments in a year (p<0.05).

| Growing season | Treatment | Yield (kg•ha^−1^) | ET (mm) | WUE (kg•ha^−1^•mm^−1^) |
| --- | --- | --- | --- | --- |
| 2018 | CDLM | 91381a | 455 | 201a |
|  | PE | 86036b |  | 189b |
|  | CK | 78093c |  | 171c |
| 2019 | CDLM | 91591a | 453 | 202a |
|  | PE | 86576b |  | 191b |
|  | CK | 77777c |  | 172c |
| 2020 | CDLM | 91621a | 458 | 200a |
|  | PE | 85960b |  | 188b |
|  | CK | 78528c |  | 171c |

**Line 371--383, Page 20:**

Film mulching techniques had significantly improved the silage corn yield with respect to no mulching [51,52]. Through our study, there are three reasons for the crop yield increase. First, although the CDLM covered field presents a little less soil temperature than PE films, the CDLM provides a more satisfactory condition for the initial crop growth than PE films, such as the low strength for seedling breaking and acceptable heat and water preservation effect. Second, the excellent degradation properties of CDLM promote the air injection to maintain soil the soil-air and increase the soil's organic matter, which PE films do not have. Third, the gradual degradation of CDLM increased the contacting of soil and air increasing water utilization on the crop roots when it rains, thus increasing crop yields [44,53]. At last, after three years of trials, it was shown that the characteristics of CDLM would further enhance the soil environment and finally promoting the growth of the crop and increasing the crop yield.

**Line 375-385, Page 20-21:**

In this work, a three-year experiment in a silage corn field was conducted in Hexi Oasis in Gansu to test the potential of CDLM to replace PE films in mulched drip irrigation. The results showed that CDLM had a heat preservation effect and promoted crop germination. The CDLM would degrade completely in ~100 days, and increase 1.01% soil organic matter in three years. Moreover, comparing to PE films, the relatively low strength is a benefit for the breaking of seedlings. The CDLM films were effective in degradation, which would reduce soil pollution generated by PE films and make a cleaner and ecological use of animal husbandry waste. The field experiment results present that the yield laid by CDLM is 6.2% and 17.2% higher than that of PE films and CK, respectively. Therefore, as an alternative to PE films, CDLM has the potential to solve the problem of waste disposal and "white pollution".

**Other changes:** We renumber the references.
